# Supplementary material for: Antifungal Peptide P852 Controls Fusarium Wilt in Faba Bean (Vicia faba L.) by Promoting Antioxidant Defense and Isoquinoline Alkaloid, Betaine, and Arginine Biosyntheses
Source: Antioxidants (Basel). 2022 Sep 7;11(9):1767. doi: 10.3390/antiox11091767 (PMC9495604; doi:10.3390/antiox11091767)
Supplement: Supplementary file 1 [file antioxidants-11-01767-s001.zip › antioxidants-1853689-SM.pdf]

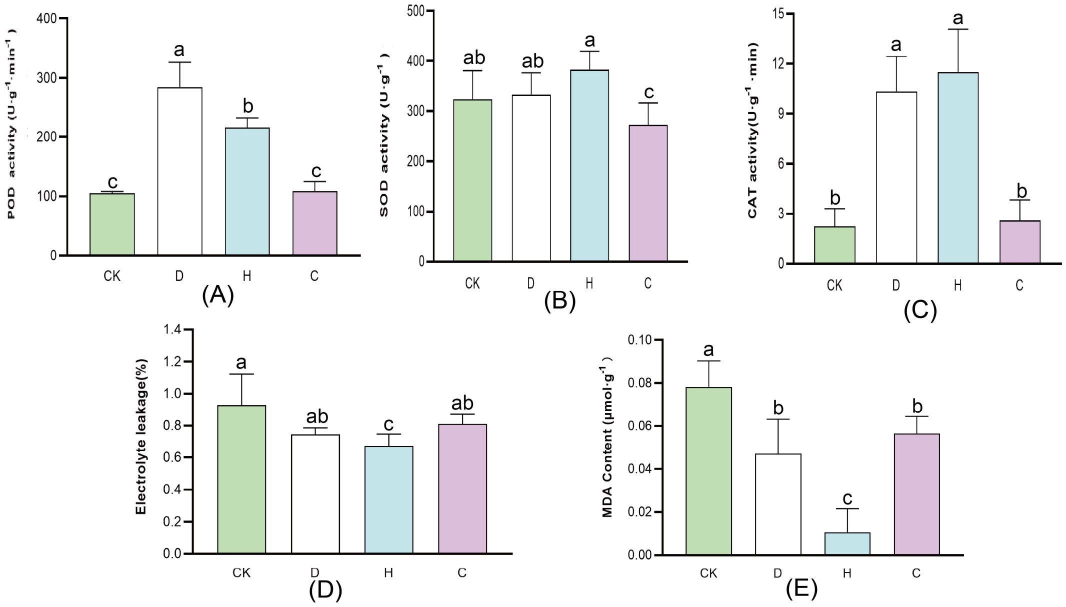

Figure S1. Effects of antifungal peptide treatments for 96 h on antioxidant capacity in the roots of faba bean that were infected with Fusarium wilt. (**A**) guaiacol peroxidase (POD) activity, (**B**) superoxide dismutase (SOD activity), (**C**) catalase (CAT) activity, (**D**) Electrolyte leakage, (**E**) MDA content. Values are means±SD (n = 3). Bars marked with different letters (a, b, c) represent significant differences ($p < 0.05$). The Fusarium wilt of faba bean were inoculated H (10 μg/mL), and D (1 μg/mL) concentrations, 10g/mL carbendazim solution(C), and seedlings treated with sterile water served as controls (CK).

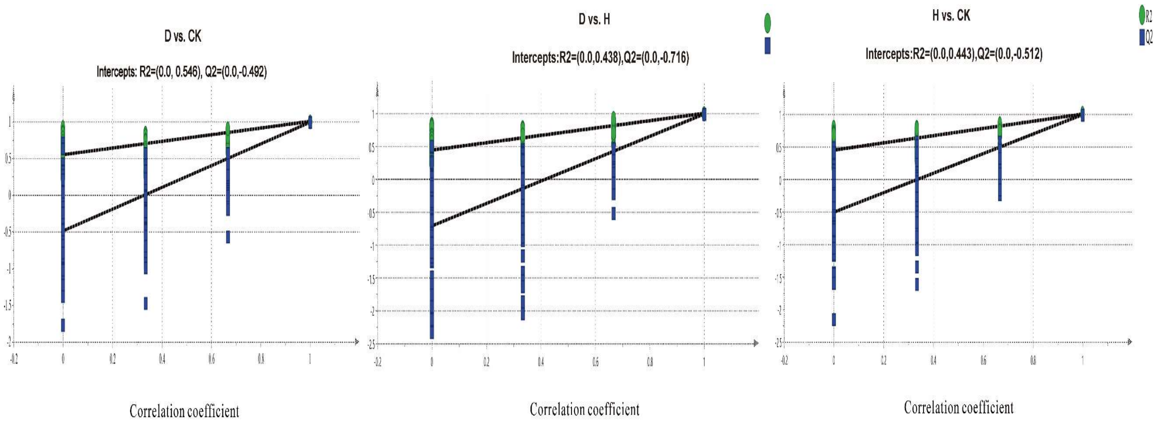

Figure S2. OPLS-DA permutation plots of the validate models in response to different treatments. H concentration treatment: P852 of 10μg/mL and D concentration treament:1μg/mL of P852 and CK: sterile water to treat Fusarium wilt the roots of Faba bean.
